# Susceptibility of the Elderly to SARS-CoV-2 Infection: ACE-2 Overexpression, Shedding, and Antibody-dependent Enhancement (ADE)

**DOI:** 10.6061/clinics/2020/e1912

**Published:** 2020-05-11

**Authors:** Jean Pierre Schatzmann Peron, Helder Nakaya

**Affiliations:** I Laboratorio de Interacoes Neuroimunes, Departamento de Imunologia - ICB IV, Universidade de Sao Paulo (USP), Sao Paulo, SP, BR; II Plataforma Cientifica Pasteur-USP, Universidade de Sao Paulo (USP), Sao Paulo, SP, BR; III Programa de Pos Graduacao em Alergia e Imunopatologia, Faculdade de Medicina FMUSP, Universidade de Sao Paulo, Sao Paulo, SP, BR

**Keywords:** SARS-CoV-2, Immunopathology, ACE-2

## Abstract

The world is currently facing a serious SARS-CoV-2 infection pandemic. This virus is a new isolate of coronavirus, and the current infection crisis has surpassed the SARS and MERS epidemics that occurred in 2002 and 2013, respectively. SARS-CoV-2 has currently infected more than 142,000 people, causing 5,000 deaths and spreading across more than 130 countries worldwide. The spreading capacity of the virus clearly demonstrates the potential threat of respiratory viruses to human health, thereby reiterating to the governments around the world that preventive health policies and scientific research are pivotal to overcoming the crisis. Coronavirus disease (COVID-19) causes flu-like symptoms in most cases. However, approximately 15% of the patients need hospitalization, and 5% require assisted ventilation, depending on the cohorts studied. What is intriguing, however, is the higher susceptibility of the elderly, especially individuals who are older than 60 years of age, and have comorbidities, including hypertension, diabetes, and heart disease. In fact, the death rate in this group may be up to 10-12%. Interestingly, children are somehow less susceptible and are not considered as a risk group.

Therefore, in this review, we discuss some possible molecular and cellular mechanisms by virtue of which the elderly subjects may be more susceptible to severe COVID-19. Toward this, we raise two main points, i) increased ACE-2 expression in pulmonary and heart tissues in users of chronic angiotensin 1 receptor (AT1R) blockers; and ii) antibody-dependent enhancement (ADE) after previous exposure to other circulating coronaviruses. We believe that these points are pivotal for a better understanding of the pathogenesis of severe COVID-19, and must be carefully addressed by physicians and scientists in the field.

## INTRODUCTION

The world is facing a major public health crisis due to the pandemic caused by a recently-described coronavirus, named SARS-CoV-2 1-3. Reaching proportions that far surpass those of SARS and MERS, the SARS-CoV-2 epidemic started in Wuhan, China in December 2019, but has now spread to more than 130 countries worldwide and has infected approximately 142,000 people, with more than 5,000 deaths being attributed to it (WHO, March 13^th^ 2020) 4. Sequencing analysis of the viral genome has revealed mutations in the spike protein—which is essential for SARS-CoV-2 attachment and invasion into host cells—may have favored the spill over from bats to humans 1. Most patients infected with coronaviruses develop a mild flu-like disease, in which the most common symptoms are fever and cough. However, in a study of 1,099 patients from 552 hospitals from 30 provinces of China in 2020, Guan W et al. 5, revealed that 15.7% of the patients who develop severe disease have increased difficulty in breathing because of pneumonia. Radiological imaging of the lungs revealed opacity in 56.4% of the patients. Approximately 2.7% of the patients needed assisted ventilation, and 1.4% died 1.

However, coronavirus disease (COVID-19) may rapidly develop into severe acute respiratory syndrome (SARS) in elderly subjects (>60 yr), especially in those with comorbidities, such as hypertension, diabetes, and pulmonary diseases 1,4,6. What is intriguing is that, unlike in the case of influenza 7, children are not included in the risk group, as very few cases of severe COVID-19 in children have been reported, and there have been no reports of death in children under the age of 9. This raises questions regarding the cellular and molecular mechanisms associated with the severity of COVID-19. Understanding and elucidating such mechanisms may greatly improve our knowledge of the pathogenesis of the disease, and thus guide health professionals as to how to better treat the elderly population.

Toward this, we raise two main points of discussion, i) the increased angiotensin-converting enzyme-2 (ACE-2) expression in pulmonary and heart tissues of hypertensive patients with chronic use of AT1R blockers and ii) antibody-dependent enhancement (ADE) after previous exposure to other circulating coronaviruses.

### SARS-CoV-2 and ACE-2

After entering the host—usually through aerosolized viral particles or contact with contaminated surfaces—the virus needs to undergo its biological cycle. Spike proteins—that are coded by the S gene in one of the open reading frames of the viral genome—need to interact with viral receptors on the surface of host cells. SARS-CoV-2 spike proteins bind to angiotensin-converting enzyme-2 (ACE-2), which is expressed in the epithelial cells of the lungs 8,9. This is the main reason why coronaviruses often cause respiratory disease. Notably, ACE-2 may also be highly expressed in intestinal tissues 9, leading to diarrhea, as observed in 60% of the patients during the SARS-CoV epidemic in 2002. Only a few patients with SARS-CoV-2 infection had diarrhea, although viral particles may be detected in the stool 10. After attaching to the ACE-2 through the receptor-binding domain (RBD) of the S1 and S2 domains of the spike protein, the viral envelope fuses with the host cell membrane and is further internalized. Genetic material, a positive RNA strand of approximately 20-32 kb, is released into the cytoplasm for replication 1. Thus, the importance of ACE-2 expression dynamics for viral infectivity, tropism, and pathogenicity is evident.

ACE-2, or ACE-related carboxypeptidase, is an 805 amino acid transmembrane protein which is an important member of the renin-angiotensin system that plays a pivotal role in the regulation of blood pressure 11. ACE converts angiotensin I (Ang I) into angiotensin II (Ang II), whereas ACE-2 converts Ang II into angiotensin 1-7 (Ang 1-7) or angiotensin 1-9 (Ang 1-9) 12. Ang II and Ang 1-7 have antagonizing effects, as Ang II binds to angiotensin 1 receptor (AT1R), inducing vasoconstriction and increase in blood pressure, whereas Ang 1-7 binds to AT2R, leading to vasodilatation and decrease in blood pressure 11,12.

As the renin-angiotensin system affects blood pressure and kidney function, ACE inhibitors and AT1R blockers are widely used in hypertensive and cardiac patients 13. In this context, the chronic use of ACE inhibitors or AT1R antagonists may be of particular relevance for patients infected with SARS-CoV-2, as they may alter the dynamics of ACE-2 expression and thus increase susceptibility to SARS-CoV-2 infection.

It has already been demonstrated by using hypertensive rat models that AT1R blockade elevates ACE-2 expression. Treatment with losartan and lisinopril, either alone or in combination, significantly increases ACE-2 mRNA in cardiomyocytes of rats. This is associated with higher Ang 1-7 plasma concentrations 14. This was corroborated by analyzing the heart tissue from rats with myocardial infarction that were treated with losartan 15. Concordantly, olmesartan, a more effective AT1R antagonist, significantly increased both cardiac and renal expression of ACE-2 in Wistar–Kyoto rats 16. This is in agreement with the use of perindopril, an ACE inhibitor, in rats 17.

Another interesting feature of the dynamics of ACE-2 expression in tissues is its ability to be cleaved from the cell surface by a metalloproteinase called ADAM17 (TACE) 18. The ACE-2 ectodomain gets cleaved, releasing soluble ACE-2 (sACE-2), whose role has not been fully elucidated. However, it has been shown that a higher concentration of sACE-2 in the plasma correlates with a poorer prognosis after heart failure 19. Notably, sACE-2 may also be detected at higher concentrations in the cerebrospinal fluid in hypertensive patients. This has been corroborated in Nefh^cre^x AT1aR^flox/flox^ mice, indicating that ACE-2 and sACE-2 levels in the brain are associated with the etiology of neurogenic hypertension 20.

Further, it was demonstrated that ACE-2 may be cleaved by other mechanisms. For instance, the SARS spike protein may modulate ADAM17 expression, which in turn cleaves ACE-2 into its soluble form. This was found to be dependent on the cytoplasmic domain of ACE-2, as siRNA against ADAM17 or ACE-2 lacking intracellular domains abrogated this phenomenon 21. Interestingly, increased shedding of ACE-2 correlates with worsening of the disease, probably because of an increase in Ang II instead of Ang 1-7. This leads to increased vascular permeability and local inflammation. Interestingly, ACE-2 cleavage from the cell surface may also occur in lung epithelial cells 22. Thus, cleavage of ACE-2 into sACE-2 would compromise the effect of Ang 1-7 on AT2R, thereby reducing pulmonary hypertension and inflammation.

**Figure 1:**
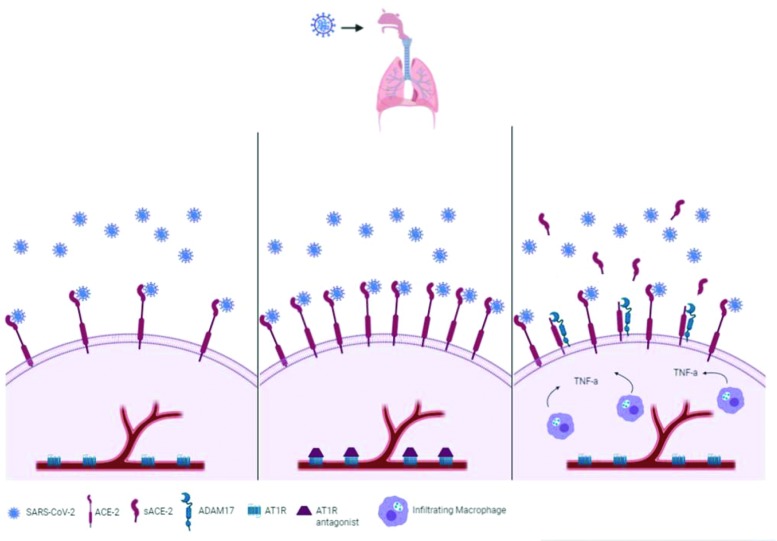
Illustrative scheme of ACE-2 expression in the lungs. Left panel: Normally expressed ACE-2 in the lung tissue interacts with SARS-CoV-2. Middle panel: Increased expression of ACE-2 in lung tissues of hypertensive patients under chronic treatment with AT1R blockers. Right panel: ADAM17 cleaves ACE-2, releasing its soluble form, sACE-2, whose levels are increased in the presence of TNF-α. Illustration was developed by the authors using www.biorender.com.

Altogether, as depicted in Figure 1, these data show that modulating the renin-angiotensin axis alters ACE-2 expression in several tissues, especially in the lung and heart tissues. The use of ACE inhibitors and AT1R antagonists seems to upregulate ACE-2 expression, facilitating viral attachment and entry. The further presence of the virus itself or some cytokines, including TNF-α, leads to ACE-2 release from the cell membrane, abrogating its function to counteract Ang II.

Altogether, ACE-2 overexpression may facilitate viral replication in lung tissue and promote lung vascular permeability, a common feature of severe SARS-CoV-2 infection. In summary, this may greatly impact the outcome of SARS-CoV-2 infection in elderly and hypertensive patients, as ACE-2 is the putative attachment and invasion receptor for coronaviruses 8,23. In fact, the use of TACE inhibitors as SARS antiviral agents has already been proposed in experimental models 2,3,24.

## ANTIBODY-DEPENDENT ENHANCEMENT

ADE is a phenomenon by which viruses use preexisting non-neutralizing antibodies from previous exposure to invade host cells through Fc receptor-mediated internalization. This is most commonly observed during secondary dengue virus (DENV) infection, causing severe hemorrhagic disease 25. Notably, the possibility of ADE between Zika virus (ZIKV), the causative agent of ZIKV congenital syndrome 26, and DENV has been intensely debated recently 27-29. Moreover, ADE has been the focus of debate for Ebola 30 and HIV 31 infections.

During ADE, preexisting antibodies elicited during a previous viral exposure are not able to neutralize viral particles during a secondary infection with any antigenic-related virus. Instead, IgG-opsonized viral particles then target FcγR expressed on endothelial and immune cells, facilitating viral internalization into host cells. Further, after intense viral replication, endothelial cells may respond by increasing vascular permeability and allowing exudate extravasation and bleeding. However, monocytes become highly activated and may initiate a cytokine storm 32.

In the context of SARS-CoV-2, it is plausible to think that ADE occurs. As mentioned in the introduction section, elderly (>60 yr) patients are more susceptible to infection for unknown reasons. As coronaviruses in general are highly prevalent in the global population, causing mild infection and flu-like symptoms, seroconversion into previous circulating coronaviruses is probably widespread. Thus, it is reasonable to infer that elderly patients, for obvious reasons, have been exposed to previous infections more times than younger subjects. This would imply a vaster repertoire of antibodies against coronavirus epitopes produced by long-living plasma cells, which in fact has recently been shown to expand during SARS-CoV-2 infection.

However, whether these antibodies are either neutralizers or enhancers must be further addressed. It is worth mentioning that, concerning the new SARS-CoV-2, sequencing analysis of the viral genome isolated in Wuhan, China indicated that mutations mainly occurred within the coding sequence of the spike protein, which has less than 40% sequence identity with that of previously circulated coronaviruses 1. These mutations may be responsible not only for the spill over from bats to humans, but also for inducing ADE, as changes in spike epitopes may result in interactions with non-neutralizing antibodies. Corroborating this, a novel epitope, which was lacking in previous isolates, was mapped 33.

As COVID-19 is not a hemorrhagic disease, it is probable that ADE, if present, is not mediated by endothelial cells. However, it has already been shown that lung epithelial cells express high levels of FcγRIIa 34. Moreover, immune cells, including monocytes and dendritic cells, highly express this receptor. These populations, especially monocytes, greatly account for the inflammatory infiltrate in the lungs during pneumonia, which is consistent with the transient lymphopenia observed in patients as circulating cells may migrate to the lungs. Conversely, lung imaging of patients with severe COVID-19 shows a great degree of lung opacity, which is consistent with edema and cellular infiltrate 5. Thus, it is feasible that lung-infiltrating monocytes expressing FcγR greatly favor SARS-CoV-2 replication in the lung tissue, accounting for the greater susceptibility of the elderly patients.

Corroborating this hypothesis, young individuals and children do not represent the risk group for severe disease, which greatly differs from the case of influenza 7, for instance. In the context of ADE, it is plausible to think that children—as they had less or no exposure to previous circulating coronaviruses—carry a very restricted repertoire of IgG—or only low-affinity IgM—which is not capable of inducing ADE. In this context, mapping complementarity determining regions (CDRs) in IgGs from young and elderly individuals would be of great importance not only to find neutralizing antibodies but also to address this hypothesis.

Additionally, previous studies on MERS and SARS have already highlighted the possibility of ADE. A recent report published by Wan Y et al. elucidated the mechanism by which monoclonal antibodies (mAbs) induce ADE in human cells. Interestingly, mAbs that target the RBD of SARS and MERS spike proteins induced conformational changes in the protein that favors an interaction with dipeptidyl peptidase 4 (DPP4), the receptor for MERS 38. Moreover, immunocomplexes also promoted viral entry. However, increasing concentrations of antibodies block viral invasion, as RBDs become inaccessible.

This is consistent with previous findings by Wang Q et al.^39^ who infected the promonocytic cell line HL-CZ—that expresses both ACE-2 and FcγR—with SARS-CoV-2 in the presence of increasing concentrations of anti-sera. Their data demonstrated that high concentrations of antibodies neutralized the virus, whereas lower concentrations induced ADE. More interestingly, the anti-sera also recognized spike protein-related antigens.

Another study on SARS-CoV-2 using Rhesus monkeys showed that immunization with full-length SPIKE glycoprotein led to increased disease severity, mostly to because of an increase in neutralizing antibodies (NAb) 35. The study demonstrated that NAbs switched the phenotype of lung-infiltrating macrophages to a pro-inflammatory M1 profile, instead of the tissue-healing profile M2. This aggravated lung injury and greatly contributed to its pathology. Conversely, previous studies using samples from deceased patients suffering from SARS-CoV infection indicated that NAb titers reached higher levels earlier in these patients, compared to that in patients who survived 36. This may indicate that ADE and the role of preexisting antibodies are in fact very relevant to the overall outcome of the infection.

**Figure 2:**
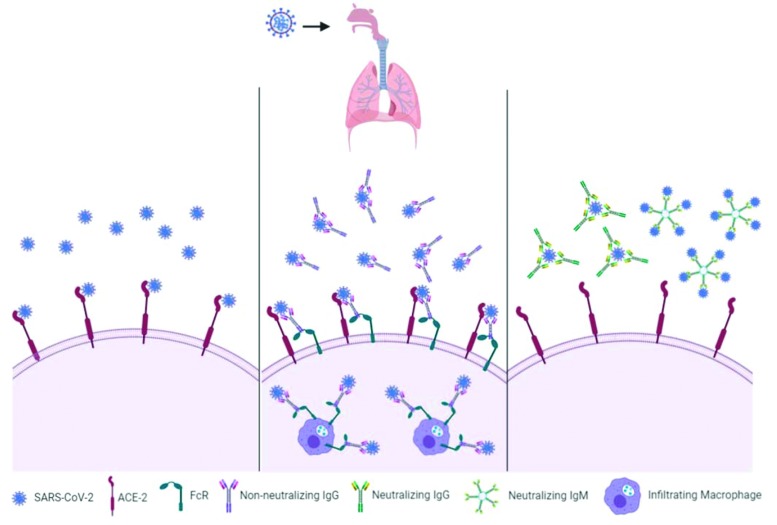
Illustrative scheme of ADE during SARS-CoV-2 infection. Left panel: First infection and lack of preexisting antibodies allow viral particles to interact with ACE-2. Middle panel: Preexisting low-affinity antibodies or antibodies at sub-optimal concentrations bind to viral particles and facilitate the viral internalization mediated by FcRs expressed on either the epithelial or immune cells. Right panel: Neutralizing IgGs elicited in response to vaccination, or neutralizing IgM that do not mediate enhancement bind to viral particles. Illustration created by the authors using www.biorender.com.

Thus, as indicated in Figure 2, circulating antibodies, instead of neutralizing the current circulating SARS-CoV-2, may bind to viral particles and thus promote Fc-mediated internalization by lung epithelial cells and infiltrating monocytes, contributing to the worsening of COVID-19. These are the most recent and mechanistic studies on ADE of coronaviruses thus far. Although the data are consistent, whether the phenomenon of ADE is observed in patients with severe COVID-19 is yet to be determined. Noteworthy, however, is the fact that immune complexes of low-avidity antibodies at sub-optimal concentrations were also responsible for the worsening of the pulmonary disease caused by H1N1 during the 2009 epidemic 37.

## CONCLUSIONS

SARS-CoV-2 is the newest threat to human health and needs emergency action from governments and public health agencies worldwide, especially because of its pandemic potential as recently declared by the World Health Organization (WHO). In this context, it is essential to rapidly and deeply address all the possibilities concerning the severity of infection, especially in the elderly population that accounts for approximately 10-12% of the mortality rate. Here, we present several relevant aspects that may contribute to the increased susceptibility of the aforementioned population to COVID-19. We believe that i) increased expression of ACE-2 in hypertensive patients being treated with ACE inhibitors and AT1R blockers and ii) previous exposure to circulating coronaviruses with low neutralizing capacity to SARS-CoV-2 may greatly contribute to the increased susceptibility of the elderly patients to COVID-19. To determine whether these hypotheses are correct, further investigations are needed, not only to better understand the etiology of the current SARS-CoV-2 infection, but also to be better prepared for future epidemics.

## AUTHOR CONTRIBUTIONS

Peron JPS conceived and wrote the manuscript. Nakaya H helped editing the manuscript.
